# Hepatic Immune Microenvironment in Alcoholic and Nonalcoholic Liver Disease

**DOI:** 10.1155/2017/6862439

**Published:** 2017-08-09

**Authors:** Jin-Seok Byun, Hyon-Seung Yi

**Affiliations:** ^1^Department of Oral Medicine, School of Dentistry, Kyungpook National University, Daegu 41566, Republic of Korea; ^2^Research Center for Endocrine and Metabolic Diseases, Chungnam National University School of Medicine, Daejeon 35015, Republic of Korea; ^3^Department of Internal Medicine, Chungnam National University Hospital, Daejeon 35015, Republic of Korea

## Abstract

Many types of innate (natural killer cells, natural killer T cells, and Kupffer cells/macrophages) and adaptive (T cells and B cells) immune cells are enriched within the liver and function in liver physiology and pathology. Liver pathology is generally induced by two types of immunologic insults: failure to eliminate antigens derived from the gastrointestinal tract which are important for host defense and an impaired tissue protective tolerance mechanism that helps reduce the negative outcomes of immunopathology. Accumulating evidence from the last several decades suggests that hepatic immune cells play an important role in the pathogenesis of alcoholic and nonalcoholic liver injury and inflammation in humans and mice. Here, we focus on the roles of innate and adaptive immune cells in the development and maintenance of alcoholic liver disease and nonalcoholic fatty liver disease/nonalcoholic steatohepatitis. Additionally, the pathogenesis of liver disease and new therapeutic targets for preventing and treating alcoholic liver disease and nonalcoholic fatty liver disease/nonalcoholic steatohepatitis are discussed.

## 1. Introduction

**Figure 1:**
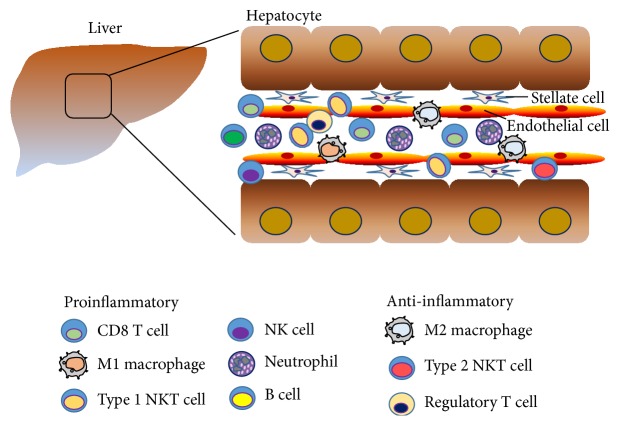
*Diverse types of hepatic immune cells in the development of liver diseases*. In the liver, actions and interactions of immune cells with hepatocytes are important for the maintenance of tissue homeostasis as well as the pathogenesis of alcoholic or nonalcoholic liver diseases.

The liver receives a dual blood supply from the hepatic artery and portal vein carrying nutrients and endotoxins from the gastrointestinal tract [1], which is closely associated with many types of immune cells, such as lymphocytes, natural killer (NK) cells, natural killer T (NKT) cells, monocytes, macrophages, and eosinophils (Figure 1) [1, 2]. Although these immune cells are involved in host defense by pathogen clearance and antigen presentation to lymphocytes [3], hepatic immune cells are also involved in the pathogenesis of metabolic and fibrotic liver diseases via interactions with hepatocytes, sinusoidal endothelial cells, or hepatic stellate cells. Among hepatic lymphocytes, approximately 50% of whole liver lymphocytes are composed of innate lymphocytes, including NK, NKT cells, and *γδ* T cells [1, 4, 5]. Liver resident macrophages, known as Kupffer cells (KCs), account for 20% of nonparenchymal cells in the liver; they are involved in the innate immune response, leading to pathogen clearance by enhancing phagocytic activity [6]. Infiltrating monocytes, accounting for approximately 5% of nonparenchymal cells, have been implicated in hyperglycemia in mice [7]. Moreover, eosinophils, comprising 1-2% nonparenchymal cells, are known to activate liver regeneration by secreting interleukin- (IL-) 4 [8]. High-fat diet feeding and alcohol consumption also alter the composition of immune cells in the liver [9, 10]. Taken together, under physiological and pathological conditions, immune cells interact with adjacent cells and secret cytokines, leading to the development of alcoholic and metabolic liver disease. Therefore, in this review, we summarize the role of each immune cell in alcoholic and nonalcoholic liver disease and hepatic fibrosis.

## 2. Alcoholic Liver Diseases

### 2.1. NK Cells

NK cells are innate immune cells involved in the development of alcoholic liver disease as well as in antiviral and antitumor immunity. Acute ethanol consumption inhibits NK cell activity in vivo, thereby promoting tumor metastasis [9, 11]. Chronic alcohol consumption impairs the immune surveillance and cytotoxicity of NK cells by arresting NK cell development at the CD27^+^CD11b^+^ stage [12, 13]. Moreover, chronic ethanol feeding decreases IL-2-mediated binding activity of NK-kB and AP-1, reducing the expression of perforin, granzyme A, and granzyme B by NK cells in mice [14]. Alcohol-induced downregulation of NKG2D, TRAIL, and interferon- (IFN-) *γ* expression has also been detected on NK cells and is associated with the activation of hepatic stellate cells [15]. NK cell numbers and cytotoxicity are also reduced in patients with alcoholic liver disease, which contributes to enhanced susceptibility to viral hepatitis and the development of hepatic fibrosis and cancer in chronic alcoholics [16]. Further studies are needed to clarify the effecter molecules that reverse NK cell cytotoxic activity to treat alcoholic liver diseases.

### 2.2. T Lymphocytes

Chronic exposure to excess ethanol induces changes in immunophenotyping in T cells from mice and humans. This leads to increased susceptibility to infections and a compromised tissue response to injury. Previous studies have revealed increased numbers of both CD4+ and CD8+ T cells in the portal and sinusoidal regions of the liver in patients with alcoholic liver disease, such as alcoholic hepatitis and cirrhosis [17]. CD62L (L-selectin adhesion molecule) is downregulated on peripheral blood lymphocytes from both humans and mice maintained on a regimen of chronic alcohol administration [18, 19]. Patients with alcoholic liver disease also show increased populations of CD4^+^CD57^+^ and CD8^+^CD57^+^ T cells in the peripheral blood. The intensity of CD44 is higher in CD8+ T cells from mice after chronic ethanol consumption compared to those from control mice [19]. In CD4^+^ and CD8^+^ T cells, a subset of CD57^+^ T cells showed higher expression levels of IFN-*γ* and tumor necrosis factor- (TNF-) *α* than those in CD57^−^ T cells, in both healthy controls and patients with alcoholic liver disease [20]. Larger amounts of IFN-*γ* and IL-4 are produced by T cells from alcohol-fed mice than controls [19]. Moreover, chronic-binge ethanol administration accelerates the loss of surface CD28 expression and promotes immunosenescence in CD8T^+^ cells from Rhesus macaques with viral diseases [21]. Furthermore, chronic alcohol administration promotes hepatic inflammation by upregulating the NF-*κ*B and STAT3 signaling pathway in a T cell-mediated hepatitis mouse model [22].

Hepatic osteopontin, which promotes the activation of macrophages and T cells, was increased in ethanol-fed mice and patients with alcoholic hepatitis, but not in alcoholics with fatty liver only [23]. Leptin stimulates the activation and proliferation of lymphocytes and promotes T-helper type 1 reactions [24]. Exposure to alcohol increases the production of leptin in adipocytes in patients with alcoholic liver disease [25]. Patients with alcoholic liver disease and ethanol-fed rodents exhibit high titers of anti-CYP2E1 autoantibodies [26], which are positively correlated with hepatic lymphocyte infiltration and increased apoptotic hepatocytes [27].

### 2.3. NKT Cells

Chronic excessive alcohol consumption induces the accumulation and activation of NKT cells in the liver, thereby promoting liver injury by recruiting neutrophils and CD11b^+^Gr-1^+^ cells [28–30] as well as increasing Fas and TNF-*α* expression in NKT cells [31]. Invariant NKT cell-deficient* Jα18* knockout mice were protected from liver injury and hepatic fat accumulation in a chronic-binge ethanol administration mouse model [28, 29]. Expression levels of various cytokines and chemokines related to inflammation and neutrophil recruitment, such as IL-1*β*, IL-6, TNF-*α*, MIP-1*β*, and CXCR1, are also increased in the livers of alcohol-fed WT mice, but not in invariant NKT cell-deficient mice [28, 29]. On the other hand, alcohol also induces the activation of NKT cells by facilitating the loading of CD1d molecules, thereby reducing the incidence of diabetes in nonobese diabetic mice [32].

NKT cells are divided into two main subsets according to differences in T cell receptor (TCR) characteristics. Semi-invariant TCR (iNKT or type 1) NKT cells are increased and activated following chronic and binge ethanol feeding in mice, and this is associated with hepatic inflammation, leading to the clinical signs of alcoholic liver disease [29]. In contrast, the more diverse TCR repertoire (type 2) NKT cells have a regulatory effect on liver injury and attenuate alcoholic liver disease in mice with chronic alcohol feeding [33]. Sulfatide-mediated activation of type 2 NKT cells modulates hepatic-recruited invariant NKT cells, thereby preventing inflammatory liver disease by inducing anergy of invariant NKT cells [34, 35].

However, the precise role of NKT cells in the pathogenesis of alcoholic liver disease should be further clarified.

### 2.4. Kupffer Cells and Hepatic Infiltrating Macrophages

Lipopolysaccharides (LPS) promote the activation of KCs, stimulating the production of inflammatory cytokines and chemokines, profibrotic molecules, and reactive oxygen species (ROS). Chronic exposure to alcohol increases the LPS concentration in the blood of humans and mice by impairing the barrier function of the intestinal mucosa [36–38]. Chronic alcohol administration induces structural alterations in gut epithelial cells of humans and mice, enhancing intestinal hyperpermeability to macromolecules [39, 40]. Higher LPS concentrations during chronic ethanol consumption promotes the activation of TLR4 and enhances production of proinflammatory cytokines in KCs [41–44]. Depletion of KCs by gadolinium chloride can protect against chronic ethanol-mediated fat deposition, inflammation, and necrosis [45, 46]. Probiotic- (*Lactobacillus subtilis* and* Streptococcus faecium*) mediated suppression of LPS-producing gram-negative bacteria markedly decreased alcohol-induced liver injury in a randomized-controlled multicenter study [47]. Taken together, these studies revealed that LPS-mediated activation of KCs is critical for the development of alcoholic liver disease.

TLR4/LPS signaling-mediated NF-*κ*B activation in KCs was enhanced in a rat intragastric feeding model of alcoholic liver disease, thereby contributing to liver necrosis and inflammation [48]. Mice with global knockout of TLR4 were protected from chronic ethanol-mediated fat accumulation and inflammation in the liver, but alcohol-fed TLR2 knockout and MyD88 knockout mice showed no differences in liver injury and steatosis compared to alcohol-fed WT mice [49]. Thus, the adaptor protein MyD88 is not essential for TLR4-mediated alcoholic liver injury and steatosis. Suppression of NF-*κ*B activation by administration of the I*κ*B suppressor gene via tail-vein injection inhibited ethanol-induced liver injury [50].

Chronic alcohol exposure to KCs also stabilized* TNF-α* mRNA, which is an important mechanism for expressing TNF-*α* [51]. NADPH oxidase, as a major contributor of ROS production, induces NF-*κ*B activation and subsequent TNF-*α* expression in KCs [52]. Inhibition of NADPH oxidase protects against alcohol-mediated oxidative liver injury by suppressing hepatic NF-*κ*B activation and TNF-*α* expression [53]. In addition, chronic alcohol consumption plays a remarkable role in phenotype changes of hepatic macrophages, including M1-proinflammatory or M2 alternative anti-inflammatory cells. Macrophages and KCs exhibit plasticity or the ability to polarize towards specific activation states and show different responses to various intra/extracellular signals [54]. Chronic alcohol intake induces an imbalance in M1/M2, leading to hepatocyte injury via hyperactivity of proinflammatory cytokines.

### 2.5. B Cells

B cells play critical functions in humoral immunity by producing antibodies and in cellular immunity by presenting antigens to T cells. Alcohol consumption also affects the lymphocyte population including B cells in the spleen and blood of mice. Chronic alcohol consumption induces lymphopenia and reduces B cells in the blood of melanoma-bearing mice but does not promote apoptosis of B cells in the blood [55]. This reduction of B cells in the blood occurs following an impaired splenocyte response to S1P by downregulating S1PR1 expression on splenocytes [55]. Moreover, ethanol exposure impairs B cell differentiation by suppressing Pax5 and IL-7R*α* expression in vitro [56]. Alcohol also inhibits antigen-induced B cell activation and proliferation, and the production of antibodies in B cells is reduced by treatment with alcohol in vitro [57]. In contrast, activation of B cells via TLR9 is associated with increased immunoglobulin A in patients with alcoholic cirrhosis [58].

### 2.6. Other Cells

Alcohol affects the function and migration of neutrophils during the development of alcoholic liver disease. Ethanol inhibits the expression of IL-8 and TNF-*α* in human polymorphonuclear neutrophils, explaining the enhanced risk of infection in alcoholic patients with nonfunctioning neutrophils [59, 60]. Intercellular adhesion molecule 1 (ICAM-1) expressed in the endothelium is a key factor in neutrophil infiltration of the liver. ICAM-1 deficiency decreased alcohol-induced liver injury and hepatic migration of neutrophils in mice fed a high-fat liquid diet with ethanol [61]. Depletion of neutrophils by injection of anti-Ly6G antibodies reduced hepatic injury in a mouse model of chronic-binge ethanol feeding. E-selectin knockout prevented the infiltration of neutrophils and liver injury in chronic-binge ethanol-fed mice, suggesting that E-selectin regulates alcohol-induced hepatic inflammation by modulating the migration of neutrophils [29]. CCL2 is also important for neutrophil recruitment in the liver. The level of CCL2 expression in the liver is correlated with disease severity in patients with alcoholic liver disease [62]. CXCL1 expression in hepatocytes and hepatic stellate cells promotes neutrophil infiltration in the liver via CXCR2 expressed on neutrophils, leading to the development of alcoholic liver injury in humans and mice [63]. The hepatocyte apoptotic index is strongly correlated with the neutrophil infiltration index in liver biopsies of alcoholic hepatitis [64].

Eosinophils may regulate macrophage polarization by producing Th2 cytokines in the liver, but the role of eosinophils and Th2 cytokines in alcohol-induced liver injury remains unclear. Increased levels of glucocorticoid in chronic heavy alcoholics [65] may be associated with defective migration and chemotaxis of eosinophils in the liver. Further studies are necessary to determine the relationship or causality between alcohol consumption and the functional properties of hepatic eosinophils in the development of alcoholic liver disease.

Although the presence of hepatic dendritic cells (DCs) is clear, the precise function of hepatic DCs remains unclear. DCs in the liver are comprised of several subsets: myeloid, lymphoid, plasmacytoid, and NK. In contrast to other resident DCs, hepatic DCs are not effective for T cell priming. Instead, they secrete cytokines in response to TLR stimulation [66]. Stimulation of TLR7 and TLR9 in hepatic plasmacytoid DCs results in the production of IFN-*α* and TNF-*α*, IL-12, or IL-6 by LPS or lipoteichoic acid via TLR4 or TLR2 in conventional DCs of the liver [67]. Hepatic DCs exacerbate or alleviate hepatocellular damage in various liver injury models by producing pro- or anti-inflammatory cytokines [68, 69]. Furthermore, alcohol consumption can modulate the functions of DCs by altering the immunophenotype and inflammatory cytokine secretion profile in DCs [70, 71]. However, the roles of DCs in the pathogenesis of alcoholic liver injury compared to other immune cells remains controversial.

## 3. Nonalcoholic Fatty Liver Disease and Nonalcoholic Steatohepatitis

### 3.1. NK Cells

NK cells play an important role in nonalcoholic fatty liver disease (NAFLD) and nonalcoholic steatohepatitis (NASH). Diet-induced obesity suppresses NK cell cytotoxic activity in rats, but energy restriction in obese rats induces the restoration of NK cell cytotoxic activity [72]. In humans, obesity reduces NK cell activity and promotes the detrimental effects of cigarette smoking compared to lean subjects [73]. Moreover, adiponectin enhances the ability of NK cells to kill target cells in lean subjects [73]. Patients with NASH show an increase in hepatic NK cells and significantly higher expression levels of NKG2D, MIC A/B, TRAIL-DR5, and CD95/Fas in liver tissues than those of healthy controls [74]. In biopsies, there were no significant differences in the mean populations of hepatic NK cells and IFN-*γ*+ NK cells between patients with NASH and hepatitis C virus-infected patients [75]. In methionine- and choline-deficient (MCD) diet-induced NASH, NKp46+DX5+ NK cells are increased in frequency and number. Depletion of NKp46+ cells aggravates NASH and increases collagen deposition in the liver [76]. NK cells are also activated by several inflammatory cytokines, including IL-12, IL-15, and IL-18, thereby contributing to IFN-*γ* production [77, 78]. These data suggest that interferon gamma (IFN-*γ*) secreted from NKp46+ NK cells inhibits the progression of NASH to fibrosis or cirrhosis.

### 3.2. T Lymphocytes

T lymphocytes, composed of T helper (Th) and cytotoxic T cells, play a central role in the pathogenesis of NAFLD/NASH and cell-mediated immunity. In a recent study, NAFLD induced by an MCD diet caused a loss of CD4+ T lymphocytes and promoted the development of hepatocellular carcinoma in inducible liver-specific* MYC* oncogene transgenic mice [79]. Moreover, depletion of intrahepatic CD4+ T lymphocytes contributed to hepatocarcinogenesis in inducible liver-specific* MYC* oncogene transgenic mice. Interestingly, C18:2 induced cell death in CD4+ T lymphocytes in vivo, suggesting that obesity-induced lipid accumulation promotes disease progression from NAFLD to hepatocellular carcinoma via the selective loss of CD4+ T lymphocytes [79]. Th17 cells and IL-17 expression were increased during progression from NAFLD to NASH in the livers of human patients and mice [80, 81]. In a mouse model of diet-induced NASH, hepatic and adipose CD4+ROR*γ*t+ T cells significantly increased, without a concurrent increase in Treg cells [82]. Hepatic regulatory T (Treg) cells were decreased by oxidative stress-mediated apoptosis of Treg cells in the livers of mice given a high-fat diet [83]. The Th17 cell/Treg cell ratio was also higher in patients with NASH compared to patients with NAFLD or healthy controls [80]. This dysregulated cellular equilibrium between Th17 cells and Treg cells is associated with the activation of Th17 cell differentiation as well as an increase in proinflammatory cytokines, including IL-6 and TNF-*α* [84, 85]. Several experimental approaches have emerged to restore this imbalance in in vivo mouse models. 3,3′-Diindolylmethane ameliorated hepatic steatosis and inflammation and restored the imbalance in Th17 cells/Treg cells in an MCD diet-induced mouse model of NASH [86]. A normocaloric low-cholesterol diet restored the balance between Th17 and Treg cells in patients with chronic hepatitis C virus infection but not in patients with NAFLD or NASH [87]. Simvastatin also decreased the expression and secretion of IL-17 in CD4+ T cells from human subjects [88].

CD8+ T cells are also associated with the mechanism underlying NAFLD and NASH. Activation of CD8+ T cells has been implicated in the development of liver cancer from NASH in mice fed a choline-deficient high-fat diet [89]. During this process, CD8+ T cells interact with hepatocytes, leading to the transition of NASH to hepatocellular carcinoma via activation of the Light*β*R and NF-*κ*B signaling pathway in hepatocytes [89]. Moreover, NF-*κ*B1 knockout mice, another NASH model, exhibited increased recruitment of hepatic CD8+ T cells, without changing the population of CD4+ T cells and NK cells in the liver [90]. These increased hepatic CD8+ T cells exacerbated liver inflammation and fibrosis in NF-*κ*B1 knockout mice. Additionally, CD8+ T cells have been implicated in the initiation and perturbation of hepatic inflammation as well as glucose intolerance in mice fed a high-fructose diet [91]. RAG1 knockout mice fed a high-fructose diet did not show lower fat accumulation in the liver compared to control mice [92]. Furthermore, CD8+ T cells are abundant in the livers of pediatric patients with NASH. The expression of IFN-*γ* is increased in the livers of patients with NASH. The population of IFN-*γ*-producing CD4+ and CD8+ T cells is also enhanced in patients with NASH [93]. Thus, hepatic CD8+ infiltrating cells and increased expression of IFN-*γ* indicate the presence of a local cytotoxic response in the livers of pediatric patients with NASH.

### 3.3. NKT Cells

NKT cells, accounting for 30% of hepatic nonparenchymal cells, are a unique subtype of lymphocytes that express surface markers of both NK and T cells [94]. NKT cells are activated by CD1d-lipid-antigen recognition, leading to hepatic inflammation and NASH [95]. Depletion of KC induced a reduction in hepatic IL-12 expression and restored the population of hepatic NKT cells in mice fed a choline-deficient diet. In addition, the high-fat diet-induced fatty liver increased the number of KCs and enhanced the expression of proinflammatory cytokines in KCs, leading to depletion of hepatic NKT cells by the overactivation and cell death of NKT cells during the development of NAFLD [96]. Moreover, adoptive transfer of NKT cells reduced hepatic fat content, subsequently causing a shift from macrosteatosis to microsteatosis and improving glucose intolerance in* ob/ob* mice [97]. The accumulation of hepatic NKT cells was observed during MCD diet-induced NASH in mice. Activation of the Hedgehog pathway mediated the recruitment and retention of NKT cells, thereby promoting myofibroblastic activation of hepatic stellate cells, leading to fibrosis progression in NASH [98]. In a human study, CD3+CD56+ NKT cells in the liver of patients with NAFLD significantly increased with increasing disease activity [99]. Hepatic CD1d expression was also enhanced in NASH patients with relatively high disease activity. Additionally, hepatic NKT cells secreted LIGHT and promoted hepatic steatosis and liver damage, finally resulting in a transition from NASH to hepatocellular carcinoma [89]. In contrast, choline-deficient diet-induced hepatosteatosis decreases the number of NKT cells in the liver in a KC- and IL-12-dependent manner [100]. Norepinephrine supplementation decreases apoptosis in hepatic NKT cells and thereby reduces Th1 cytokines and increases the production of Th2 cytokines, leading to the suppression of LPS-mediated hepatotoxicity [101]. The difference in the population and activation of NKT cells among studies of NAFLD/NASH may be related to context-specific effects of mouse models or human patients. Therefore, additional extensive studies are required to determine the role of NKT cells in the development of NAFLD/NASH.

### 3.4. KCs and Hepatic Infiltrating Macrophages

KCs are hepatic resident macrophages that participate in inflammatory signaling and metabolic fluctuations. High-fat diet-mediated impaired barrier function of intestinal mucosa increases the LPS concentration in the blood [102]. KCs were activated by continuous exposure to LPS, which resulted in the activation of Toll-like receptor 4 (TLR4) and enhanced the production of inflammatory cytokines [103]. TLR4-deficient mice are protected from high-fat diet-induced liver damage and lipid accumulation [104]. KCs are the major source of hepatic proinflammatory cytokines, such as TNF-*α* [105]. Moreover, depletion of KCs with clodronate liposomes suppresses the development of steatohepatitis and insulin resistance [104, 106]. Mice with KCs derived from MyD88 knockout bone marrow donors exhibited lower inflammation and fat accumulation in the liver [107]. During the migration of myeloid cells into the liver, CCR2 is a major chemokine receptor during NAFLD development [108]. Fat overload-mediated steatosis leads to lipid deposition in KCs, which is associated with dysregulation of lipid metabolism and the expression profile of proinflammatory cytokines in the KCs. Additionally, fat-laden KCs increase recruitment of lymphocytes, and this is reversed by the suppression of lipogenesis in KCs [54]. Treatment with short-chain fatty acids increases the expression of TLRs in murine KCs in vitro [96]. On the other hand, short-chain fatty acids inhibit the activity of proinflammatory cytokines by suppressing NF-*κ*B activation in KCs [109]. Moreover, short-chain fatty acids produced by gut microbiota contribute to the alleviation of high-fat diet-induced fat accumulation in the liver [110]. Furthermore, endocannabinoid secretion by KCs promotes hepatic lipid deposition and fibrosis in a mouse model of NAFLD/NASH [111]. However, recent studies have also shown that the depletion of hepatic macrophages exacerbates or does not induce changes in hepatic steatosis and insulin resistance in mice fed a high-fat diet [112, 113]. These discrepancies may be related to the duration or time point of KC depletion in the liver and differences in depletion methodologies or knockout mice. In streptozotocin-induced hyperglycemia, infiltrating monocytes (CD11b^+^F4/80^int^) and neutrophils were enhanced, but KCs were unchanged in the liver [7]. Infiltrating hepatic monocytes from mice with streptozotocin-induced hyperglycemia showed higher expression of TNF-*α*, IL-1*β*, IFN-*γ*, and IL-6 compared to those in control mice. Thus, infiltrating monocytes, rather than KCs, play a major role in the development of hepatic inflammation in an insulin-deficient model of mice.

### 3.5. B Cells

Recently, B cells were found to be important in the development of metabolic diseases, such as type 2 diabetes and obesity, by modulating T cells and producing antibodies [114]. However, little is known about the role of B cells in the pathogenesis of NAFLD/NASH. B cell-activating factor (BAFF), which is a superfamily member of TNF, was increased in the serum of patients with NASH compared to controls with simple steatosis [115]. Higher serum levels of BAFF are also correlated with the extent of ballooning hepatocytes and hepatic fibrosis. BAFF knockout and BAFF receptor knockout mice show reduced numbers of mature B cells [116]. BAFF knockout mice exhibit reduced systemic inflammation [117] and treatment with BAFF impairs insulin sensitivity by inhibiting the insulin signaling pathway in mice [118]. Treatment with LPS induces low levels of IL-10 but high levels of IFN-*γ*, IL-6, and TNF-*α* in hepatic B cells other than splenic B cells [119]. This finding suggests that hepatic B cells promote rather than suppress local inflammatory responses.

### 3.6. Other Cells

Neutrophils have been implicated in acute phase inflammation. Obesity induces infiltration of neutrophils into the liver, which is mediated by IL-8 and growth-related oncogene alpha, leading to the development of NAFLD/NASH [120, 121]. Infiltrating neutrophils are also enhanced and frequently surrounded by lipid-laden hepatocytes in the livers of patients with NASH [121]. Neutrophil myeloperoxidase enzyme-induced oxidative stress promotes the formation of oxidized phosphatidylcholine and progression of hepatic steatosis in patients with NASH [122], but a deficiency of myeloperoxidase reduces neutrophil infiltration, fat accumulation, and fibrotic change in the livers of low-density lipoprotein-deficient mice fed a high-fat diet [123]. Additionally, depletion of neutrophil elastase decreases the hepatic neutrophil content and the inflammatory response, accompanied by an increase in hepatic insulin sensitivity [124]. Furthermore, patients with NASH and fibrosis show a higher neutrophil-to-lymphocyte ratio compared to that of healthy controls, and this ratio is a predictive marker of systemic inflammation and advanced disease [125].

Eosinophils secrete Th2 cytokines, including IL-4 and IL-13, which promote the activation of M2 macrophages. The role of eosinophils in macrophage polarization is well-established in adipose tissue, but not in the liver. Moreover, wild-type mice show increased beige fat mass under conditions of cold exposure, whereas the depletion of eosinophils or loss of IL-4/13 signaling in mice fails to induce biogenesis of beige fat [126]. However, little is known about the functions of eosinophils and Th2 cytokines in the development of NAFLD/NASH and thus further analyses are required to elucidate the relationship between NAFLD and beige fat.

## 4. Summary and Future Perspectives

**Figure 2:**
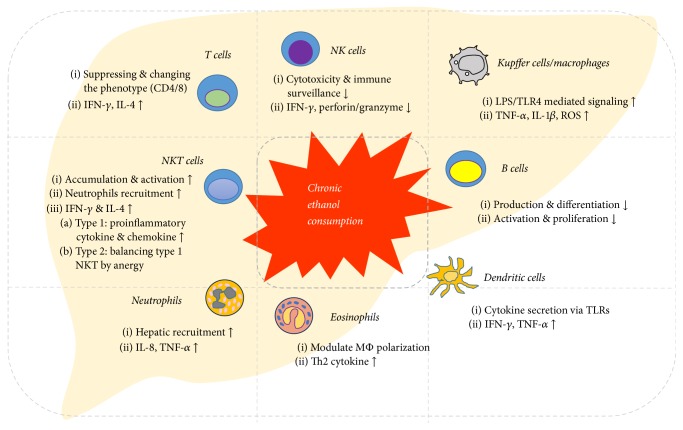
*Chronic alcohol consumption impairs the functional properties of both innate and adaptive immune cells in the liver*. Chronic alcohol intake results in the activation of innate immunity components, such as KCs/macrophages, and the inhibition of innate immunity components, such as NK and NKT cells, contributing to the pathogenesis of ALD. Moreover, adaptive immunity including T cells and B cells is altered by alcohol consumption, leading to the progression of ALD.

**Figure 3:**
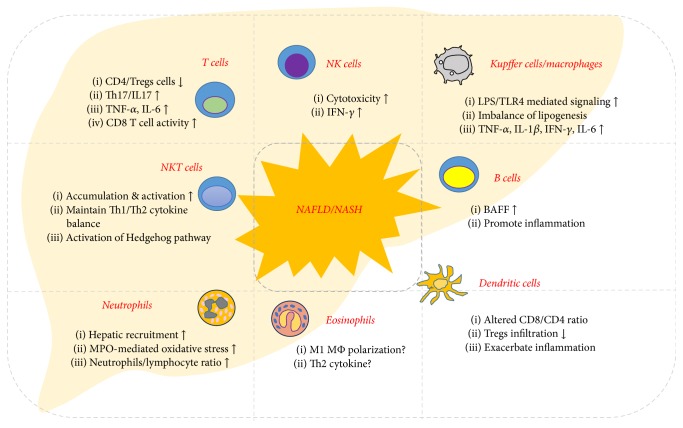
*Functional properties of both innate and adaptive immune cells in the development of NAFLD*. Diverse types of immune cells are involved in the pathogenesis of NAFLD/NASH. T and B cells, NK and NKT cells, and KCs/macrophages generally promote hepatic inflammation during the development of NAFLD/NASH. However, Treg cells and eosinophils are implicated in the improvement of NAFLD.

Hepatic innate and adaptive immune cells have been implicated in the pathogenesis of alcoholic and nonalcoholic liver disease (Figures 2 and 3). Experimental and clinical data obtained over the past few decades have indicated that they not only have beneficial effects in suppressing ALD and NAFLD/NASH, but also contribute to the exacerbation of liver injury. In general, CD8+ T cells, M1 macrophages, B cells, type 1 NKT cells, neutrophils, and NK cells secrete proinflammatory cytokines and chemokines, leading to the development of ALD and NAFLD/NASH. In contrast, M2 macrophages, type 2 NKT cells, and regulatory T cells appear to be associated with protection against liver injury.

Diverse hepatic immune cells, such as NK cells, NKT cells, KCs/macrophages, B and T lymphocytes, Th17 cells, neutrophils, eosinophils, and even dendritic cells, are influenced by alcohol, leading to various interactions with hepatic stellate cells after alcohol exposure. Chronic ethanol consumption suppresses the cytotoxicity and immune surveillance of NK cells, leading to decreased IFN-*γ*, perforin, and granzyme production. B cells are also inactivated by alcohol exposure. In contrast, chronic ethanol consumption increases the cytotoxicity of NKT and T cells, which produce high levels of IFN-*γ* and IL-4. KCs/macrophages and dendritic cells are also activated by alcohol. In particular, KCs/macrophages are mainly responsible for excessive inflammation induced by ethanol via LPS/TLR4-mediated TNF-*α* production. Alcohol increases hepatic recruitment of neutrophils and the production of IL-8 and TNF-*α* and induces Th2 cytokine-mediated macrophage polarization in eosinophils.

Various diet-induced animal models of NAFLD/NASH are used to elucidate the roles of hepatic innate and adaptive immune cells and their interactions. The activity of NK and NKT cells is decreased in a diet-induced NAFLD/NASH animal model. Moreover, Th17 cells are involved in the progression of NAFLD/NASH in humans and mice, but Treg cells protect against NAFLD/NASH via the secretion of anti-inflammatory cytokines. KCs/macrophages and B cells are activated during the development of NAFLD/NASH. Neutrophils and dendritic cells exacerbate inflammation, while eosinophils and Th2 cytokines alleviate liver injury by suppressing excessive immune responses.

A great deal of progress has been made in the mechanistic understanding of the functional properties of hepatic immune cells, and thus therapeutic modalities aimed at modulating hepatic inflammation should be developed to prevent or treat ALD and NAFLD/NASH. Therefore, prospective clinical studies are required to determine the critical role of the immune response in the progression of ALD and NAFLD/NASH. These studies will also help identify patients with ALD or NAFLD/NASH for patient-specific therapy in the age of precision medicine.
